# *Hoya isabelchanae* Rodda & Simonsson, a new, showy species of *Hoya* R.Br. (Apocynaceae, Asclepiadoideae) with pomegranate red flowers from Sulawesi, Indonesia

**DOI:** 10.3897/phytokeys.68.8803

**Published:** 2016-08-02

**Authors:** Michele Rodda, Nadhanielle Simonsson Juhonewe

**Affiliations:** 1 The Herbarium, Singapore Botanic Gardens, 1 Cluny Road, 259569 Singapore; 2 Research affiliate at National Research Institute of Papua New Guinea; PO Box 1–524, Ukarumpa, EHP 444, Papua New Guinea

**Keywords:** Borneo, Cultivation, Gunung Boliohutu, Malesia, Marsdenieae

## Abstract

A new species of *Hoya* R.Br. from Sulawesi (Indonesia), *Hoya isabelchanae* Rodda & Simonsson, is described and illustrated. It is one of the largest flowered species in Hoya section Acanthostemma (Blume) Kloppenb. Its flowers are of comparable size to those of *Hoya benchaii* Gavrus et al., *Hoya kloppenburgii* T.Green, *Hoya rundumensis* (T.Green) Rodda & Simonsson and Hoya sigillatis T.Green ssp. sigillatis, all from Borneo. Among Sulawesi species it is compared with the vegetatively similar *Hoya brevialata* Kleijn & van Donkelaar and *Hoya pallilimba* Kleijn & van Donkelaar.

## Introduction

The *Hoya* R.Br. diversity of Sulawesi (Indonesia) was investigated rather comprehensively by Kleijn and van Donkelaar (2001) who supported their herbarium studies with extensive field investigations throughout Sulawesi and not only collected herbarium specimens but also made extensive collections of sterile plants for growing *ex situ*. They estimated that Sulawesi might have up to 20 species and provided a description for eight species, three of which are new, *Hoya brevialata* Kleijn & van Donkelaar, *Hoya myrmecopa* Kleijn & van Donkelaar and *Hoya pallilimba* Kleijn & van Donkelaar. The new species were all based on specimens bloomed in cultivation in Wageningen (Netherlands). Cultivation has long been considered an essential step in the identification of *Hoya* species, that otherwise rarely bloom in the wild (Lamb et al. 2014; Rintz 1978), and that are difficult to study from exsiccates alone. In 2004 a further new species from Sulawesi was named *Hoya tomataensis* T.Green & Kloppenb., and in 2010 the element identified as *Hoya camphorifolia* Warburg by Kleijn and van Donkelaar (2001: 467–468) was named *Hoya paulshirleyi* T.Green & Kloppenb. Both species were described based on cultivated plants.

A further sterile plant collected in Sulawesi by Steve Scott and brought into cultivation at the Royal Botanic Garden Edinburgh is regarded to represent a new species and it is here described as *Hoya isabelchanae* Rodda & Simonsson.

## Species treatment

### Hoya isabelchanae

Taxon classificationPlantaeGentianalesApocynaceae

Rodda & Simonsson sp. nov.

urn:lsid:ipni.org:names:77156823-1

Figs 1 , 2

#### Diagnosis.

Among Sulawesi *Hoya* species similar to *Hoya brevialata* and *Hoya pallilimba* in habit (prostrate and pendant epiphyte), lamina shape (convex round to elliptic) and inflorescence type (positively geotropic, convex) but separated because both *Hoya brevialata* and *Hoya pallilimba* have smaller flowers (c. 5 vs. 8–10 mm in diameter in *Hoya isabelchanae*) with a finely pubescent corolla (vs. setose corolla in *Hoya isabelchanae*).

#### Type.

Indonesia, Sulawesi, Gorontalo, Gunung Boliohutu, 400 m, 23 Apr 2002, S.M. Scott 02-116, grown on at the Royal Botanic Garden Edinburgh (Acc no. 20021229), Sep 2012, *C.E. Berthold 0013* (holotype: E; isotype: SING)

#### Description.

Epiphytic climber with white latex in all vegetative parts. *Stems* slender, prostrate, pendant, internodes (2)4–6(–10) cm × 1–1.5 mm, dull green or brown, pubescent when young, rarely almost glabrous when mature; *adventitious root* sparsely produced along the stem and just under the nodes where they are usually paired. *Leaves* petiolate; petiole recurved, round, 4–6(–8) × ca. 1.5 mm, pale green, pubescent; *lamina* orbicular-ovate (to elliptic), convex, fleshy and stiff (1.5–)2–4(–7) × (1–)1.5–2.5 cm, base cuneate (round), apex obtuse (round), pale to mid-green green above, with or without grey spots, pubescent on young leaves only, paler green underneath, pubescent; penninerved, secondary veins obscure; *colleters* (one) two at each lamina base, triangular to ovate 0.2–0.4 × 0.3–0.5 mm. *Inflorescence* positively geotropic, pseudo-umbellate, slightly concave; *peduncle* (1–)2–4(–7) cm × 1.5–2 mm in diameter, dull green to brown, pubescent when young; rachis indeterminate. *Flowers* 10–15 each inflorescence; *pedicel* variable in length, the internal ones ca. 8 mm long, the external ones 2.5–3 cm × 1.2–1.5 mm in diameter, bright green, glabrous. *Calyx* lobes triangular, 1.2–1.6 × 1–0.8 mm wide, apex round, light green or brownish, glabrous; *basal colleter* one in each calyx lobe sinus, ovate, 300–400 × ca. 100 µm. *Corolla* revolute, 8–10 mm in diameter, ca. 16 mm when flattened; *corolla lobes* basally fused, tube 3–4 mm long, pomegranate red, from almost glabrous at the base to thinly pubescent becoming setose towards the distal part of the inner side of the tube, glabrous outside, lobes broadly ovate, 5–6 × 4–5 mm, pomegranate red with a paler edge, inside setose with a glabrous tip, outside glabrous. *Corona* staminal, 7–8 mm in diameter, 3–3.5 mm high; *corona lobes* ovate, ca. 3.5 × 2 mm, slightly convex above, underneath sulcate, inner process apex acuminate, outer process apex divided in an upper round part and a lower bifid part, upper part cream yellow with a pinkish inner process tip, lower part and bilobed outer process reddish. *Anthers* with apical translucent appendages, broadly triangular, c. 1.2 × 1.2 mm. *Pollinia* oblong, 250–300 × 130–150 µm, base obliquely truncate, apex round, sterile edge all along the outer edge of the pollinium; *corpusculum* oblong, 120–140 × 50–60 µm; *caudicle* broad, spathulate, 180–200 × 100 µm at the widest point. *Style-head* 5 angled in cross section, c. 2.5 mm in diameter, with 5 lobes alternating with the stamens, style-head apex mamillate, ca. 1 × 0.5 mm broad at the base; *ovary* ovoid, shortly beaked, 1.5–1.7 mm long, each carpel ca. 0.7 mm wide at the base, pale green, glabrous. *Fruit* and *seed* not observed.

#### Etymology.

*Hoya isabelchanae* is named after Isabel Claire Chan Yuen Ching, late daughter of Elisabeth Chan, Singaporean patron of botanical research and a gardener with an interest in *Hoya*.

#### Distribution and ecology.

*Hoya isabelchanae* is only known from the base of Gunung Boliohutu, Sulawesi, where it was collected as a sterile cutting in 2002 and brought into cultivation at the Royal Botanic Garden Edinburgh where it regularly blooms in a heated greenhouse from May to October. The species was collected in primary forest and it was growing in shaded but exposed area on a decaying tree 12 m tall. A further collection is widely available in cultivation under Gerard Paul Shirley number GPS10161 and 7-35 http://www.paulshirleysucculents.nl/shop_hoyas.htm [accessed on 24 June 2016]. This accession is apparently also from Sulawesi but no further collection information is available.

#### Conservation status.

The only localised specimen of *Hoya isabelchanae* is the type collection. No information is available on the extent of the wild population in Sulawesi and the threats to its habitat therefore its conservation status is Data Deficient (DD) (IUCN 2014). *Hoya isabelchanae* is at present in cultivation at the Royal Botanic Gardens Edinburgh (type collection, acc. no. 20021229) and at the Singapore Botanic Gardens (unlocalised collection, vouchered as M. Rodda MR573).

#### Notes.

**Figure 1.**
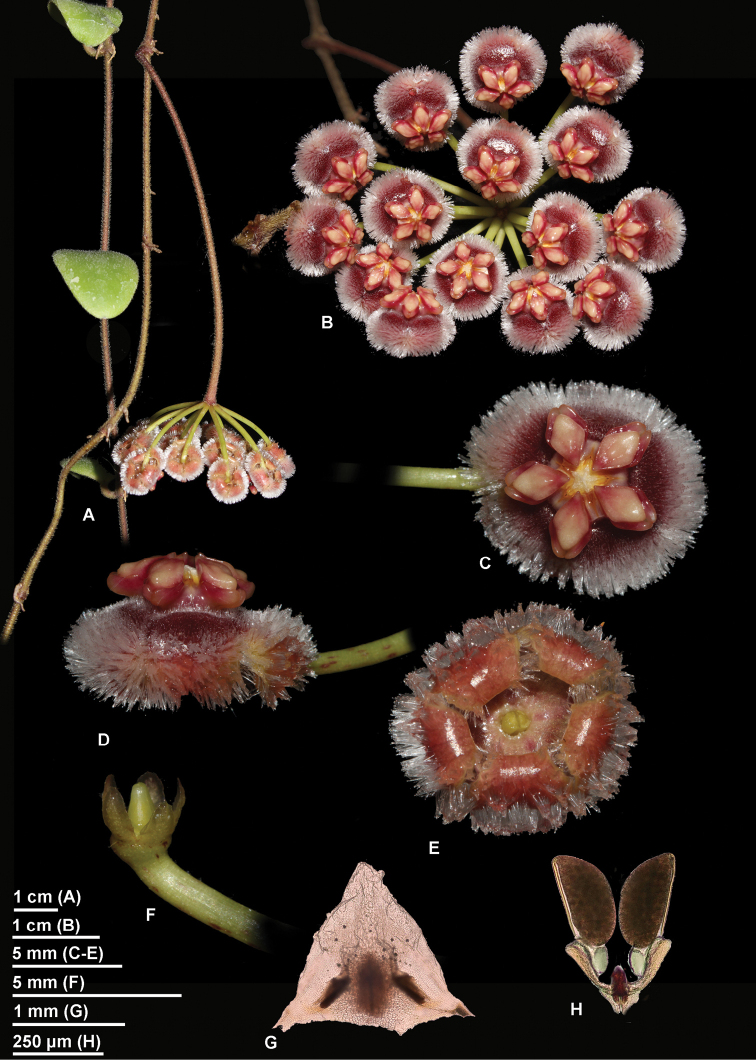
*Hoya isabelchanae* photographed in cultivation in Thailand (reference specimen *M. Rodda MR573*, SING). **A** Branch and inflorescence, side view **B** Inflorescence, from underneath **C** A single flower, front view **D** A single flower, lateral view **E** Corolla from underneath with calyx removed **F** Calyx **G** Anther **H** Pollinarium. (Photographs by M. Rodda).

*Hoya isabelchanae* belongs to Hoya section Acanthostemma (Blume) Kloppenburg whose members have revolute corolla lobes, bilobed outer corona lobes and pollinaria with broad, spathulate caudicles. With a corolla 8–10 mm in diameter, *Hoya isabelchanae* is one of the largest flowered species in *Acanthostemma*, only comparable with *Hoya benchaii* Gavrus et al. (corolla 9–12 mm in diameter) *Hoya kloppenburgii* T.Green (10–12 mm), *Hoya rundumensis* (T.Green) Rodda & Simonsson (7–10 mm) and Hoya sigillatis T.Green ssp. sigillatis (7–10 mm), all from Borneo. All these can be separated from *Hoya isabelchanae* because their corolla is puberulent while *Hoya isabelchanae* has a setose corolla (Fig. 1).

**Figure 2.**
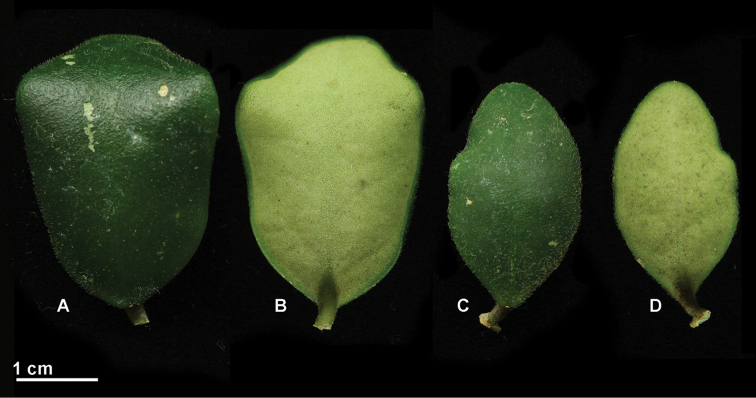
Leaves of *Hoya isabelchanae* photographed in cultivation in Singapore (reference specimen *M. Rodda MR573*, SING). **A** Larger leaf, from above **B** Larger leaf, from underneath **C** Smaller leaf, from above **D** Smaller leaf, from underneath (Photographs by M. Rodda).

Among Sulawesi *Acanthostemma* members, *Hoya isabelchanae* is vegetatively similar to *Hoya brevialata* and *Hoya pallilimba*, that make large clumps of prostrate and pendant stems and have convex round to elliptic laminas. They also have similar positively geotropic convex inflorescences. However *Hoya isabelchanae* can be separated from *Hoya brevialata* and *Hoya pallilimba* because it has much larger flowers, both *Hoya brevialata* and *Hoya pallilimba* have flowers c. 5 mm across while *Hoya isabelchanae* has flowers 8–10 mm in diameter. The corona of *Hoya isabelchanae* has almost flat lobes while the coronas of *Hoya brevialata* and *Hoya pallilimba* have the inner lobe held much higher than the outer lobe process. Further, the corolla of *Hoya brevialata* and *Hoya pallilimba* is finely pubescent while the corolla of *Hoya isabelchanae* is setose.

#### Additional specimens examined.

Indonesia, Sulawesi, (live collection numbers 7-35 and GPS10161), grown in Thailand, Ratchaburi, 23 Mar 2014, M. Rodda MR573 (SING).

## Supplementary Material

XML Treatment for Hoya isabelchanae
